# Epidemiology, clinical and physiological manifestations of dust lung disease in major industrial centers

**DOI:** 10.1186/s12982-022-00111-0

**Published:** 2022-04-07

**Authors:** Alla Philippova, Raisa Aringazina, Gulnara Kurmanalina, Vladimir Beketov

**Affiliations:** 1 Department of Biology and General Genetics, I.M. Sechenov First Moscow State Medical University, Trubetskaya str., 8-2, 119991 Moscow, Russian Federation; 2 Department of Internal Diseases No. 1, Non-Commercial Joint-Stock Society, West Kazakhstan Marat Ospanov Medical University, Maresiev str., 68, 030012 Aktobe, Kazakhstan; 3 Department of Internal Diseases No. 2, Non-Commercial Joint-Stock Society, West Kazakhstan Marat Ospanov Medical University, Maresiev str., 68, 030012 Aktobe, Kazakhstan; 4 Department of Internal, Occupational Medicine and Rheumatology, Clinical Medicine Institute, I.M. Sechenov First Moscow State Medical University, Trubetskaya str., 8-2, 119991 Moscow, Russian Federation

**Keywords:** Occupational diseases, Dust lung diseases, Pneumoconiosis, Occupational chronic bronchitis, Chronic obstructive pulmonary disease, Epidemiology

## Abstract

**Background:**

The present study aims to determine the structure of morbidity in workers contacting industrial aerosols, assess the timeliness of diagnosing dust-induced lung disease in major industrial centers, and optimize diagnostics for early detection of occupational lung diseases in workers exposed to industrial dust hazards.

**Methods:**

The study on the structure and incidence of occupational lung diseases was carried out in 2016–2020 based on the Moscow Centre for Occupational Pathology data. For a more in-depth clinical examination, 114 patients who were first admitted to the Occupational Pathology Centre with diagnosed pneumoconiosis (PC), chronic dust-induced bronchitis (CDB), and chronic obstructive pulmonary disease (COPD) were selected. All patients were subjected to a complex clinical-functional, spirographic, echocardiographic, fibroscopic, radiological, and CT lung examination, with subsequent analysis of the results obtained. The pathology caused by exposure to industrial aerosols within the studied period was first diagnosed in 344 workers. Most patients (64%) with newly detected pathologies were 50–59 years of age, with work experience in adverse conditions of 21–25 years (41%).

**Results:**

The spirographic study of respiratory function revealed decreased forced vital capacity (FVC) indices in CDB and COPD patients. Changes in expiratory flow rates suggest occupational bronchitis at an earlier stage, whereas no apparent results were noted for the PC diagnosis. The results of fibroscopic examination in PC patients revealed atrophic processes of the bronchial mucosa in 46 (88.5%) of them, and 6 (11.5%) patients had a subtropic process. The results of echocardiographic examination allowed diagnosing pulmonary heart disease in 83 patients (72.8%). Of them, 42 (80.8%) were revealed in the group of patients with PC, 18 (50.0%) in the COB group, and 14 (53.8%) in the COPD group.

**Conclusions:**

Computed tomography (CT) detected pathological changes in 52 patients, while the X-ray examination in six people showed no evidence of lung destruction. CT scan also showed that the number of patients with fibrotic PC (including silicosis) in the study groups increased. Timely clinical and functional examination (spirography, fibroscopy, echocardiography) of patients allows detecting PC (including silicosis), CDB, and COPD at an early stage of disease progression.

## Introduction

Lung diseases related to environmental problems are becoming more relevant and socially important [1]. Long-term exposure to industrial aerosols reduces the efficiency of protective mechanisms of bronchopulmonary lung segment, leading to occupational dust-induced and chronic pulmonary toxicity of lungs in workers of some industries [2, 3]. Dust-induced lung diseases are now regarded as one of the most common occupational diseases in humans. They are among the highest in terms of days of incapacity for work, disability, and death globally [4–6]. Pulmonary lesions due to prolonged exposure to dust particles of various fibrogenicity are also prominent in the structure of occupational morbidity in industrial regions of the Russian Federation. Furthermore, they cause extensive economic damages due to the loss or reduction in employees’ working capacity [7]. Thus, illnesses from exposure to industrial aerosols occupy third place in the structure of occupational pathology in the Russian Federation and the Republic of Kazakhstan, in particular in the Aktobe region and the city of Aktobe, where there are many enterprises of the oil and gas, mining and chemical industries [8]. The problem is exacerbated by the fact that in the Aktobe region many emissions of industrial enterprises into the atmosphere are carried out with insufficient purification. Thus, according to the data of the regional sanitary and epidemiological service, the presence of pollutants is detected in 50.7% of the air, while in some cases there are exceedances of maximum permissible concentrations of sulfur dioxide, nitrogen dioxide, formaldehyde, suspended solids in 5.7% [8]. A more frequent incidence is reported for diseases resulting from physical factors of production processes and physical overload of specific organs and body systems of workers [9]. In the structure of primary nosological forms of occupational pathology due to the effect of industrial aerosols in Russian Federation, chronic dust-induced bronchitis (CDB) represents 22.45%, pneumoconiosis (PC)—21.78%, chronic obstructive (asthmatic) bronchitis (COB)—18.36%, other diseases—37.41% [10]. According to Rospotrebnadzor, foundry, mining, mechanical engineering, and construction are potentially dangerous industrial areas. Potentially dangerous occupations include miners, coal miners, metallurgists, cement manufacturers, electrical welders, maintenance workers [11].

According to medical statistics in Aktobe region up to 25% of applications for medical care are related to respiratory diseases. As of 2020 in the region and Aktobe city there are higher rates of bronchitis morbidity per 100 thousand people than in the Republic of Kazakhstan as a whole both among adults and adolescents. Thus, in Aktobe region there were 389 cases of bronchitis per 100 thousand adults and 587 cases among adolescents, this figure for the Republic of Kazakhstan as a whole is 272 and 408 cases respectively [12].

Adverse production factors frequently aggravate exposure to industrial dust. They include microclimate, heavy physical labor, unfavorable environment (mainly general atmospheric pollution in industrial centers), age, upper respiratory tract infections, genetic predisposition, and smoking, which intensifies the effect of industrial aerosols [13].

At the same time, within the overall polyclinic network, diagnosis of occupational diseases due to exposure to industrial aerosols remains quite rare. Most occupational pathologies are detected only when workers seek medical care [14]. Such a proportion of independent calls for occupational diseases indicates insufficient medical examination within mandatory corporate worker quotas [15]. The early stages of these diseases are not detected during regular medical tests. In general, they are already diagnosed in severe forms, resulting in a loss of working ability and disability [16]. In recent years, occupational morbidity in the Russian industrial regions has increased quantitatively and is becoming more severe in terms of nosology. Thus, there are growing cases of neglected dust-induced severe diseases [17]. These lung diseases are characterized by the irreversibility of the course, leading to disability, reduced life quality, and life expectancy of patients [18]. There is no effective method for treating dust-induced lung diseases, which could stop its constant progression [19–21]. However, by excluding timely exposure to harmful occupational factors and applying appropriate therapy, stabilization of the disease process and disease progression is possible [22, 23]. Hence, enhancing medical methods and the quality of diagnostics for prevention, early detection, and timely treatment of patients in the initial stages of diseases are the main tasks for protecting their health [24]. It is necessary to optimize the results evaluation criteria in modern clinical and instrumental studies of early disease stages.

The present study aimed to determine the structure of morbidity in workers contacting industrial aerosols and assess the timeliness of diagnosis of dust-induced diseases in a large industrial center. Besides, consideration was given to optimizing diagnostics for early detection of occupational lung diseases in workers exposed to dust hazards.

## Materials and methods

The study on the structure and frequency of occupational lung diseases was conducted based on the Moscow City Center and Aktobe City Center for Occupational Pathology materials in 2016–2020. The dynamics of early-detected (‘primary’) occupational lung diseases following exposure to industrial aerosols during the specified period were evaluated.

Clinical examination enrolled 114 patients admitted to the Centre for Occupational Pathology for the first time and matched the following selection criteria:

1. The diagnosis of PC, moderate COB (dust-induced and toxic dust-induced) in remission, II-degree COBP without exacerbation;
2. No concurrent conditions (tuberculosis, bronchiectasis, pulmonary neoplasms); and
3. Consent to be included in the study.

The exclusion criteria were:

1. Smoking;
2. Stages II and III hypertension;
3. Coronary heart disease;
4. Circulatory insufficiency of IV functional class (FC);
5. Comorbid conditions; and
6. Use of angiotensin-converting enzyme (ACE) inhibitors and ß-Adreno-blockers.

The average age of the examined patients was 56.4 ± 7.32 years. The average time spent working under adverse conditions was 21.5 6.87 years. The majority of the patients studied were aged 50–59 years, i.e., the most skilled workers of pre-retirement age, with more than 20 years of working experience in contact with industrial aerosols.

All patients examined were allocated to three nosological groups. The first group enrolled 52 patients diagnosed with PC and silicosis. The second group comprised 36 patients diagnosed with dust-induced and toxic dust-induced bronchitis. Finally, the third group included 26 COPD patients.

The nosological features of patients by sex, age, and work experience in hazardous working conditions are presented in Table 1.

**Table 1:** Nosological features of patients by sex, age, and duration of service under hazardous working conditions

	Nosological groups	Number of people	Males	Females	Average age	Average work experience
1	PC	52	43	9	55.4 ± 6.24	20.3 ± 7.45
2	COB	36	27	9	56.4 ± 6.65	22.4 ± 6.47
3	COPD	26	15	11	57.1 ± 6.57	22.6 ± 6.27

PC, Pneumoconiosis; COB, chronic occupational bronchitis; COPD, chronic obstructive pulmonary disease

Typical symptoms of the above pathologies, such as dyspnea at rest and after exercises, cough, chest pain, and increased fatigue, were investigated to evaluate the patients’ clinical status.

All patients underwent complex clinical and functional examination, bronchoscopy, radiology, and a pulmonary tomography examination. Pulmonary ventilation function was assessed by computer spirography on the Custo Vit device (Germany). Prior to the examination, all active-acting medications on the smooth muscle tone of the bronchi were canceled. A bronchodilatation test with fenoterol hydrobromide was used to assess the reversibility of bronchial obstruction. The study was carried out in the morning, on an empty stomach, and in conditions of relative rest (sitting) using nasal clips to exclude the effect of circadian rhythm on the result.

An echocardiographic study was performed on the Hewlett Pascard Sapos-1000 (Japan) device by standard technique: M-mode and two-dimensional mode from the left parasternal position near the tips of the mitral valve leaflets.

The fibrobronchoscopic examination was carried out with the FV-ZS Olumpus (Japan) bronchoscope under local anesthesia (1% lidocaine solution). Afterward, the state of the bronchial tree and trachea was visually assessed.

Chest radiography was performed according to the standard procedure. The Valsalva test modified by Kuznetsov was applied to distinguish vascular and fibrous changes of the lungs if required. The severity of the coniotic process was estimated based on the nature of the pathological changes detected, their shape, size, profusion and prevalence in the lung fields. Small changes were distinguished as nodular shadows (“p”, “q”, “r”), linear thrusts (“s”, “t”, “u”), and large changes “A”, “B”, “C”; fibrous changes in pleura and lung roots, diffuse changes as pleural overlay (thickening) and local changes were marked as “a”, “b”, “c” plaques.

The quality of the chest radiographs obtained was evaluated using the following criteria:

1. Completeness of coverage of the studied object;
2. Correct positioning of the patient during imaging;
3. The clarity of the radiograph;
4. The contrast of the radiograph; and
5. The stiffness of the radiograph.

CT scans of the thoracic organs were performed using a 32 slice Toshiba Aquilion Multi 32 slice (Japan) CT system. Patterns, i.e., pathology features, were used to evaluate CT scans. Thus, the nodular pattern implies small nodules with a diameter ranging from 1 to 10 mm. Linear pattern includes thin irregular lines, thin linear densities, and reticulated linear densities. Data on the prevalence of pathological processes allowed distinguishing between thickening of the axial interstitium, interfollicular and intradollicular septa, the sign of “honeycombs” (combination of small cavities less than one centimeter formed by alveolar destruction with delimiting fibrous interstitial bands), subpleural lines, and centrilobular star points.

Statistical analysis of the study results was performed with the specialized software SPSS 21. Prior to research, the law of quantitative indices distribution was investigated using Kolmogorov–Smirnov and Shapiro–Wilk criteria. Thus, the distribution of the studied indices was close to the normal values, with no significant outliers, asymmetry, and kurtosis indices. Comparisons of independent groups with various forms of lung pathology were made using a univariate analysis of variance (ANOVA); comparisons of individual groups with each other were made using the Dunn criterion. For multiple comparisons, the Bonferroni type adjustment was used. The contingency tables were analyzed with the calculation of χ^2^ criterion. For two-dimensional contingency tables, statistical significance was assessed using Fisher’s exact test. Descriptive statistics used in this paper are arithmetic mean (M) and standard deviation (s)—M ± s. The critical value of the significance level (p) was taken to be 0.05.

## Results

### Occupational lung diseases due to exposure to industrial aerosols for 2016–2020

**Fig. 1:**
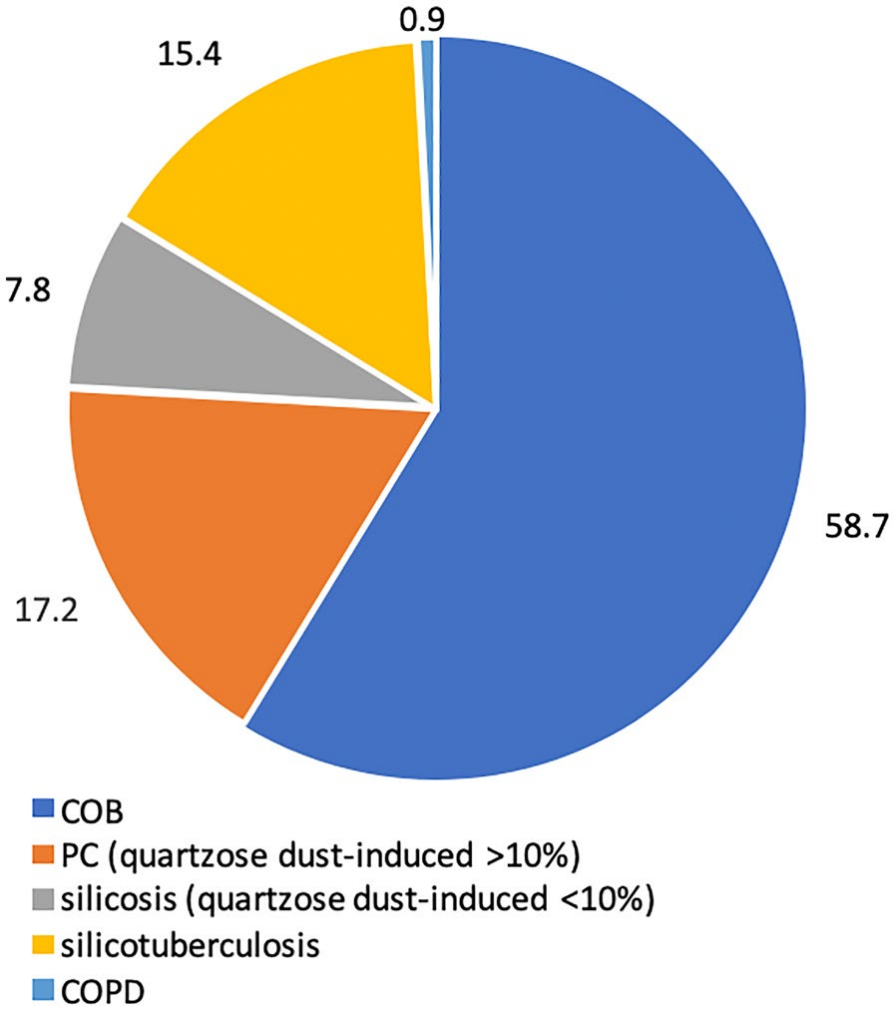
Structure of newly detected occupational diseases resulting from exposure to industrial aerosols for 2016–2020

According to the Moscow City Center and Aktobe City Center for Occupational Pathology data for 2016–2020, diseases caused by exposure to industrial aerosols were first identified in 344 workers. Thus, COB was detected in 202 (58.7%) patients, PC (quartzose dust-induced > 10%) in 59 (17.2%), silicosis (quartzose dust-induced < 10%) in 27 (7.8%), silicotuberculosis in 3 (0.9%), and COPD in 53 (15.4%) patients (Fig. 1).

Trends in the structure of occupational dust pathology first detected in 2016–2020 are relatively stable across years (Table 2).

**Table 2:** Occupational pathology due to exposure to industrial aerosols according to the Centre for Occupational Pathology in 2016–2020

Seq. No.	Nosology	Number of patients detected in different years					
		2016	2017	2018	2019	2020	Total
1	Chronic bronchitis	44	37	48	32	41	202
2	Pneumoconiosis	12	14	8	11	14	59
3	Silicosis	6	4	5	5	7	27
4	Silicotuberculosis	1	0	0	1	1	3
5	COPD	12	9	8	13	11	53
Total		75	64	69	62	74	344

**Fig. 2:**
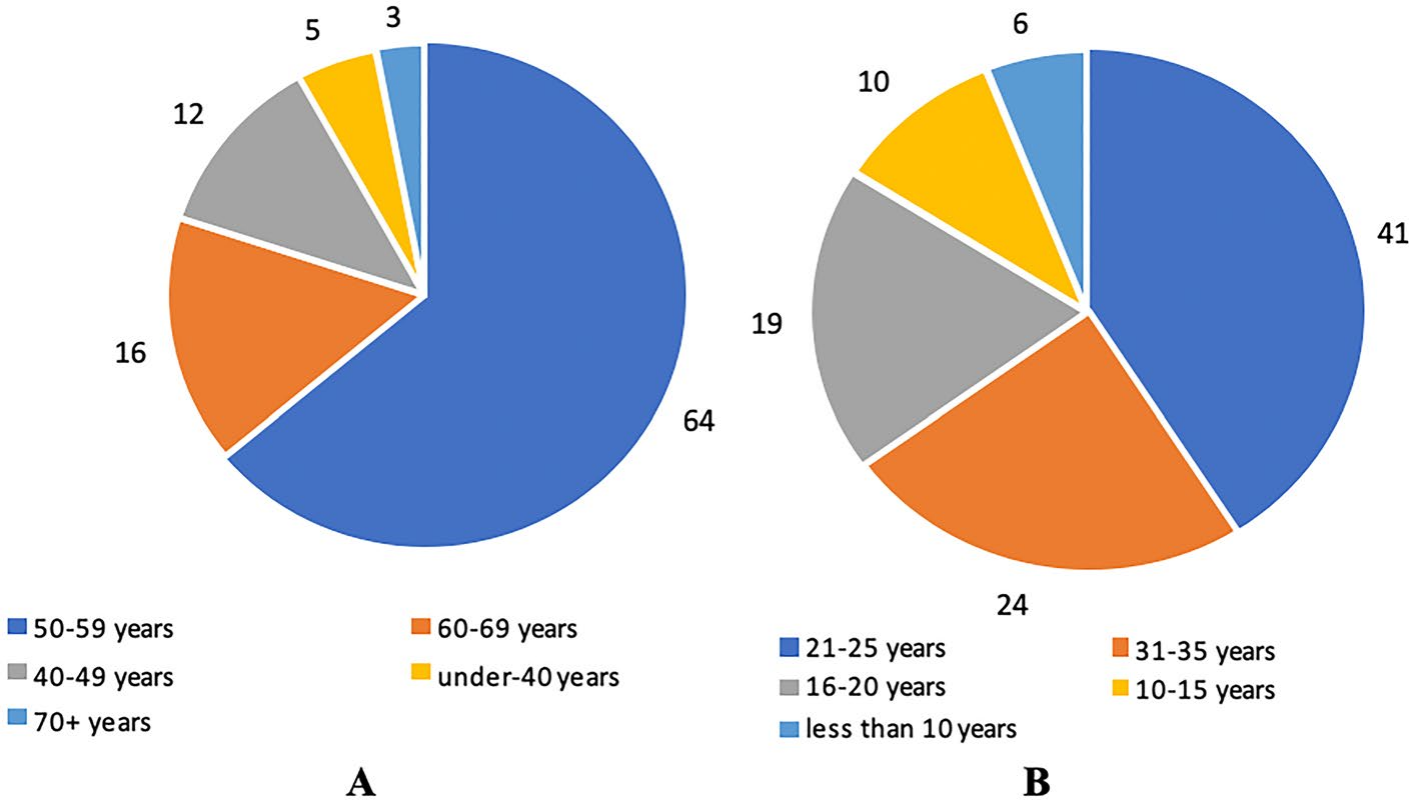
Distribution of patients with newly diagnosed occupantional diseases caused by exposure to industrial aerosols in 2016–2020 by age group (**A**) and work experience with dust hazards (**B**)

The majority of patients (64%) with newly detected conditions ranged from 50 to 59 years of age. Patients in the 60–69 age group accounted for 16%, the 40–49 age group for 12%, the under-40 age group for 5%, and the 70+ age group for 3% (Fig. 2A).

Depending on the experience of working in harmful conditions under exposure to occupational dust, patients were distributed as follows. Those with experience of 21–25 years accounted for 41%, with experience of 31–35 years for 24%, followed by 19% with experience of 16–20 years and 10% with experience of 10–15 years. Less than 10 years had 6% patients (Fig. 2B).

**Fig. 3:**
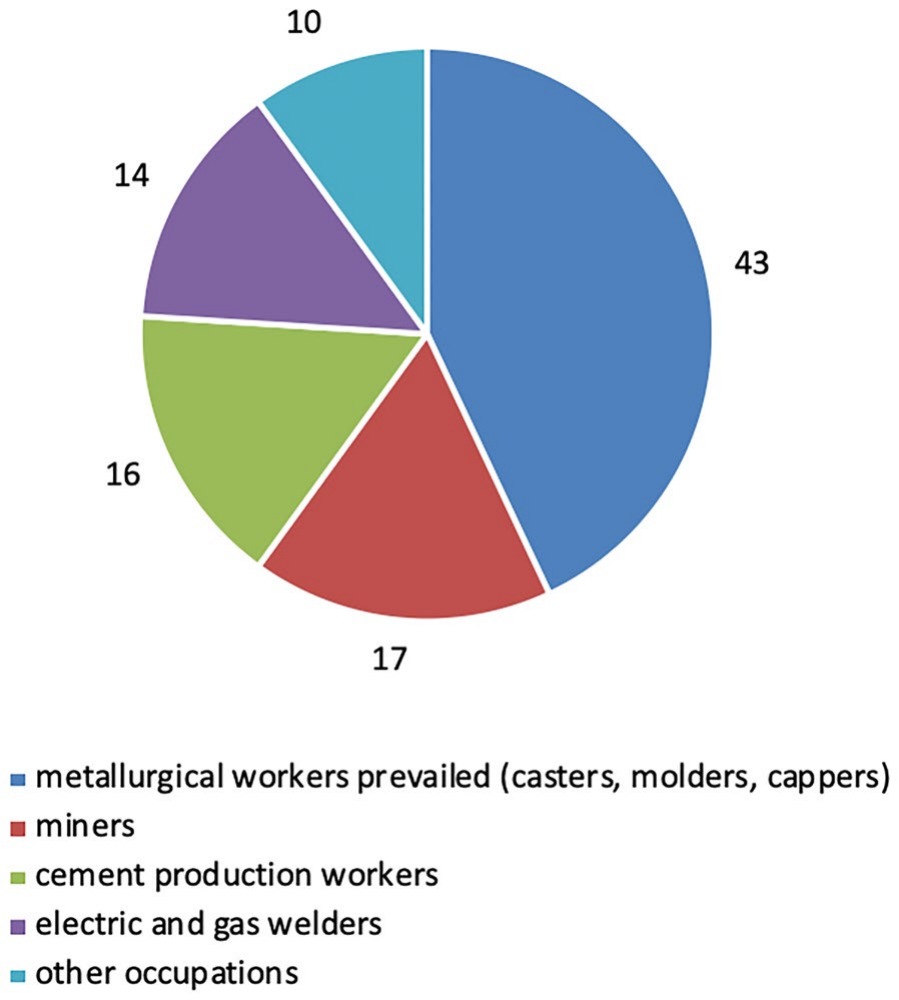
Distribution of patients with newly diagnosed occupational diseases caused by exposure to industrial aerosols by occupation in 2016–2020

By occupation, metallurgical workers prevailed (casters, molders, cappers), accounting for 43%. Miners comprised 17%, cement production workers—16%, electric and gas welders—14%, other occupations occupied 10% (Fig. 3).

### Clinical symptomatology of occupational dust-induced lung diseases

Clinical symptomatology of occupational dust lung diseases of the examined patients is presented in Table 3.

**Table 3:** Detectability of clinical signs

Symptoms	PC and silicosis	Dust-induced CB	COPD	p 1–2	p 1–3	p 2–3
Dyspnea at rest	0 (0%)	4 (11.1%)	9 (34.6%)	0.342	0.001	0.026
Dyspnea at excersing	33 (64.5%)	26 (72.2%)	17 (65.4%)	0.711	0.865	0.743
Dry cough	21 (40.4%)	21 (58.3%)	16 (61.5%)	0.121	0.012	0.314
Cough with sputum	19 (36.5%)	6 (16.7%)	6 (23.1%)	0.066	0.740	0.332
Chest pain	9 (17.3%)	2 (5.6%)	6 (23.1%)	0.223	0.436	0.015
Increased fatigue	49 (94.2%)	33 (91.7%)	18 (69.2%)	0.567	0.001	0.002
Rhinopharyngolaryngitis	13 (23.5%)	22 (61.1%)	7 (26.9%)	0.001	0.564	0.023
Box-tone	18 (34.6%)	20 (55.6%)	9 (34.6%)	0.057	0.864	0.237
Attenuated breathing	4 (7.7%)	5 (13.9%)	2 (7.7%)	0.657	0.765	0.675
Harsh breathing	24 (46.2%)	32 (88.9%)	16 (61.5%)	0.001	0.387	0.002
Stertor on forced exhalation	27 (51.9%)	28 (77.8%)	21 (80.8%)	0.013	0.067	0.865

p ≤ 0.05—differences are statistically significant; p > 0.1—differences are not statistically significant; 0.1 > p > 0.05—differences are found at the level of the statistical trend

PC, Pneumoconiosis; COB, chronic occupational bronchitis; COPD, chronic obstructive pulmonary disease

Patients diagnosed with pneumoconiosis and silicosis presented with no dyspnea at rest, dyspnea occurring during physical activity was observed in 64.5%. Dry cough complaints were registered in 40.4%, cough with sputtering in 36.5%, chest pain was registered in 17.3%, and increased fatigue was observed in 94.2% of patients.

In the group of patients diagnosed with dust-induced COB, dyspnea at physical exercise was reported by 72.2%. Dry cough was observed in 58.3%, cough with sputum in 16.7%, and chest pain in only 5.6%. Increased fatigue was noted in 91.7% of patients.

In the group of patients diagnosed with COPD, dyspnea at rest was observed in 34.6%, and dyspnea only during physical activity was noted in 65.4% of patients. Dry cough was bothering for 61.5%, and cough with sputum for 23.1%. Chest pain was reported by 23.1% and increased fatigue by 69.2% of patients.

Thus, in all three groups of patients, the main complaints were dyspnea during physical exercise, increased fatigue and cough, more often dry.

According to the physical examination data, percussion and a box tone were observed in 41.2% of patients: 34.6% in Group 1, 55.6% in Group 2, and 34.6% in Group 3. On auscultation, attenuated breath sounds were heard in 9.6% of patients (7.7% in Group 1, 13.9% in Group 2, and 7.7% in Group 3); harsh breathing was observed in 63.2% of patients (46.2% in Group 1, 88.9% in Group 2, and 61.5% in Group 3). Dry rales during forced exhalation were noted in 66.7% of patients (51.9% in Group 1, 77.8% in Group 3, and 80.8% in Group 3).

Symptoms of rhinopharyngolaryngitis were noted in 23.5% of patients with PC (20.8%). There are also upper respiratory tract symptoms in 61.1% of dust-induced COB patients and 26.9% of COPD patients.

Thus, in all three groups of dust-induced diseases, the most frequent signs were increased fatigue, shortness of breath on exertion, harsh breathing, and dry rales. According to patients’ complaints, anamnesis, and physical examination, the characteristics of PC, COB, and COPD clinics are similar.

### Results of spirographic, echocardiographic, and fibroscopic examination of patients with dust-induced occupational lung diseases

Further clinical examination involved 114 patients with a primary diagnosis of the occupational respiratory disease, who met the selection criteria. Depending on the diagnosis, these patients were divided into three groups: patients diagnosed with PC (group 1), patients diagnosed with COB (group 2), and patients diagnosed with COPD (group 3).

Spirography results in the group of patients with PC detected reduced FVC of lungs at the level of 87.4 ± 7.6% when assessing external respiratory function. Decreased FVC up to 77.5 ± 8.3% was noted in patients with COB. FVC in patients with bronchitis was lower than in those diagnosed with PC (p < 0.05). In patients with occupational COPD, there was a significant decrease of FVC index to 62.8 ± 6.4% (statistical difference with PC and CB patients at p < 0.05).

In patients with PC, the forced expiratory volume in the first second (FEV1) comprised 86.7 ± 8.8%, and the FEV1/FVC ratio, also called the Tiffeneau-Pinelli index, was 94.7 ± 9.6%, indicating restrictive changes. A downward trend was observed in FEV1 values in patients with COB (77.8 ± 8.7%, p < 0.05 compared to PC patients). A more significant decrease in FEV1 was found in COPD patients, amounting to 61.3 ± 6.1% (p < 0.05). FEV1/FVC was 82.6 ± 8.2% (p < 0.05) in COB patients and 58.3 ± 10.6% (p < 0.05) in COPD patients.

In the group of patients with PC, the maximum volume rate at the level of 25% of forced vital capacity of lungs (MEF25%) was 78.4 ± 7,1%. Significant decrease of MEF25% was noted in COB with 62.7 ± 7.7% (p < 0.05) and COPD patients with 51.4% ± 8.3% (p < 0.05). Peak expiratory flow rate (PEFR) in patients with PC was registered at 73.8% ± 9.4%. For patients with COB and COPD, this value was 66.3 ± 6.8% (p < 0.05) and 48.4 ± 7.3% (p < 0.05), respectively (Table 4).

**Table 4:** Indicators of external respiration function (average values) and changes in the volume flow rate in patients (% of the norm)

	Nosological groups	FVC, %	FEV1, %	FEV1/FVC, %	MEF25%	PEFR, %
1	PC (n = 52)	87.4 ± 7.6	86.7 ± 8.8	94.7 ± 9.6	78.4 ± 7.1	73.8 ± .4
2	COB (n = 36)	77.5 ± 8.3	77.8 ± 8.7	82.6 ± 8.2	62.7 ± 7.7	66.3 ± 6.8
3	COPD (n = 26)	62.8 ± 6.4	61.3 ± 6.1	58.3 ± 10.6	51.4 ± 8.3	48.4 ± 7.3

Significance of intergroup differences p 1–2, p 1–3, p 2–3—p < 0.05

PC, Pneumoconiosis; COB, chronic occupational bronchitis; COPD, chronic obstructive pulmonary disease. FVC, forced vital capacity; FEV1, forced expiratory volume 1-s; FEV1/FVC, Tiffeneau-Pinelli index; MEF 25%, maximum expiratory flow rate at 25% of vital capacity; PEFR, peak expiratory flow rate

According to spirographs in 52 patients diagnosed with PC and silicosis, respiratory failure was not revealed (RF 0) in 5 patients (9.6%), I-degree respiratory failure (RF I) was revealed in 31 patients (59.6%), and II-degree respiratory failure (RF II) was recorded in 16 patients (30.8%). In 36 COB patients, RF 0 was registered in 8 persons (22.2%), RF I in 23 (63.9%), and RF II in 5 (13.9%). In 26 COPD patients, RF 0 was observed in 3 patients (11.5%), RF I in 15 (57.7%), and RF II in 8 (30.8%) (Table 5).

**Table 5:** Degree of respiratory failure in examined patients

	Nosological groups	RF 0	RF I	RF II
1	PC (n = 52)	5 (9.6%)	31 (59.6%)	16 (30.8%)
2	COB (n = 36)	8 (22.2%)	23 (63.9%)	5 (13.9%)
3	COPD (n = 26)	3 (11.5%)	15 (57.7%)	8 (30.8%)

PC, pneumoconiosis; COB, chronic occupational bronchitis; COPD, chronic obstructive pulmonary disease; RF, respiratory failure

**Fig. 4:**
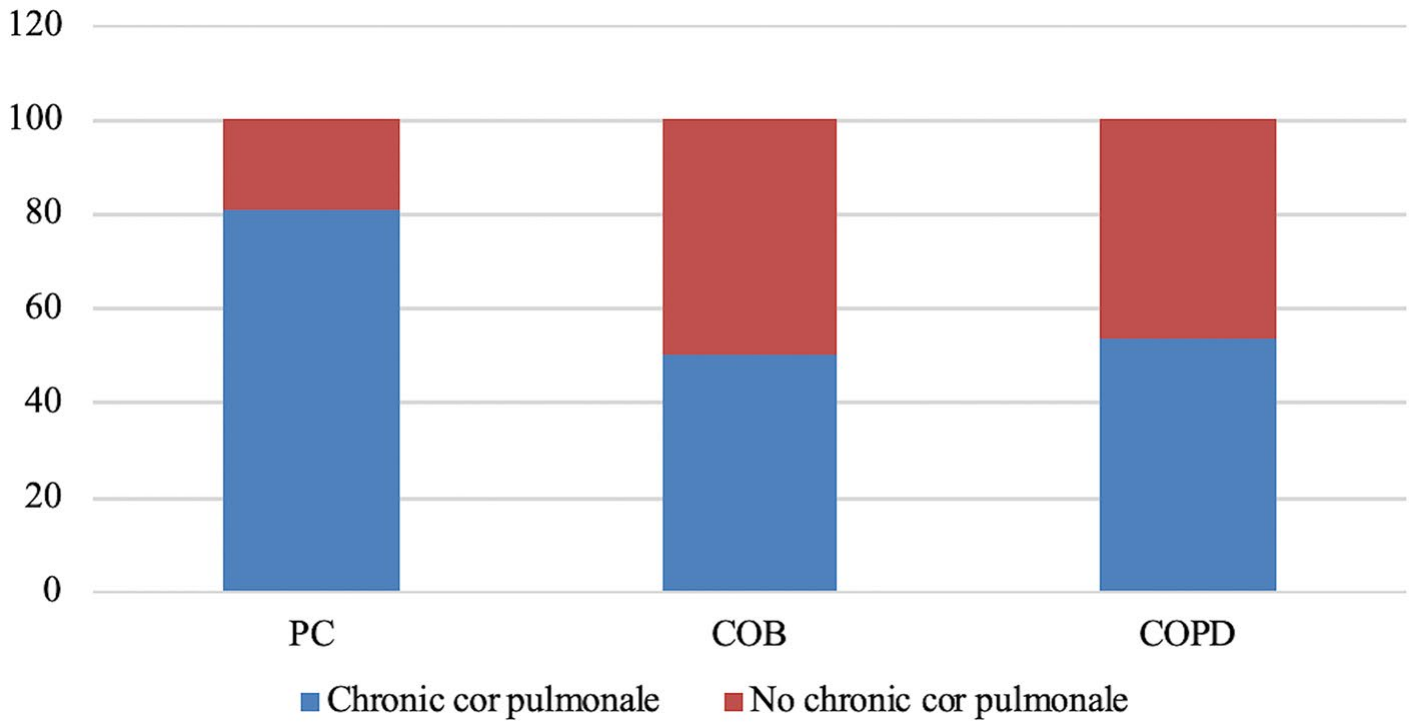
Echocardiographic signs of cor pulmonale in the examined patients (%). PC, pneumoconiosis; COB, chronic occupational bronchitis; COPD, chronic obstructive pulmonary disease

Complex analysis of clinical data and echocardiographic findings allowed diagnosing chronic pulmonary heart disease in 83 patients (72.8%). Of them, 42 (80.8%) were from PC group, 18 (50.0%) represented COB patients, and 14 (53.8%) had COPD. At the same time, decompensated pulmonary heart disease (with different decompensation degrees) was observed in 53.6% across all groups (Fig. 4).

**Fig. 5:**
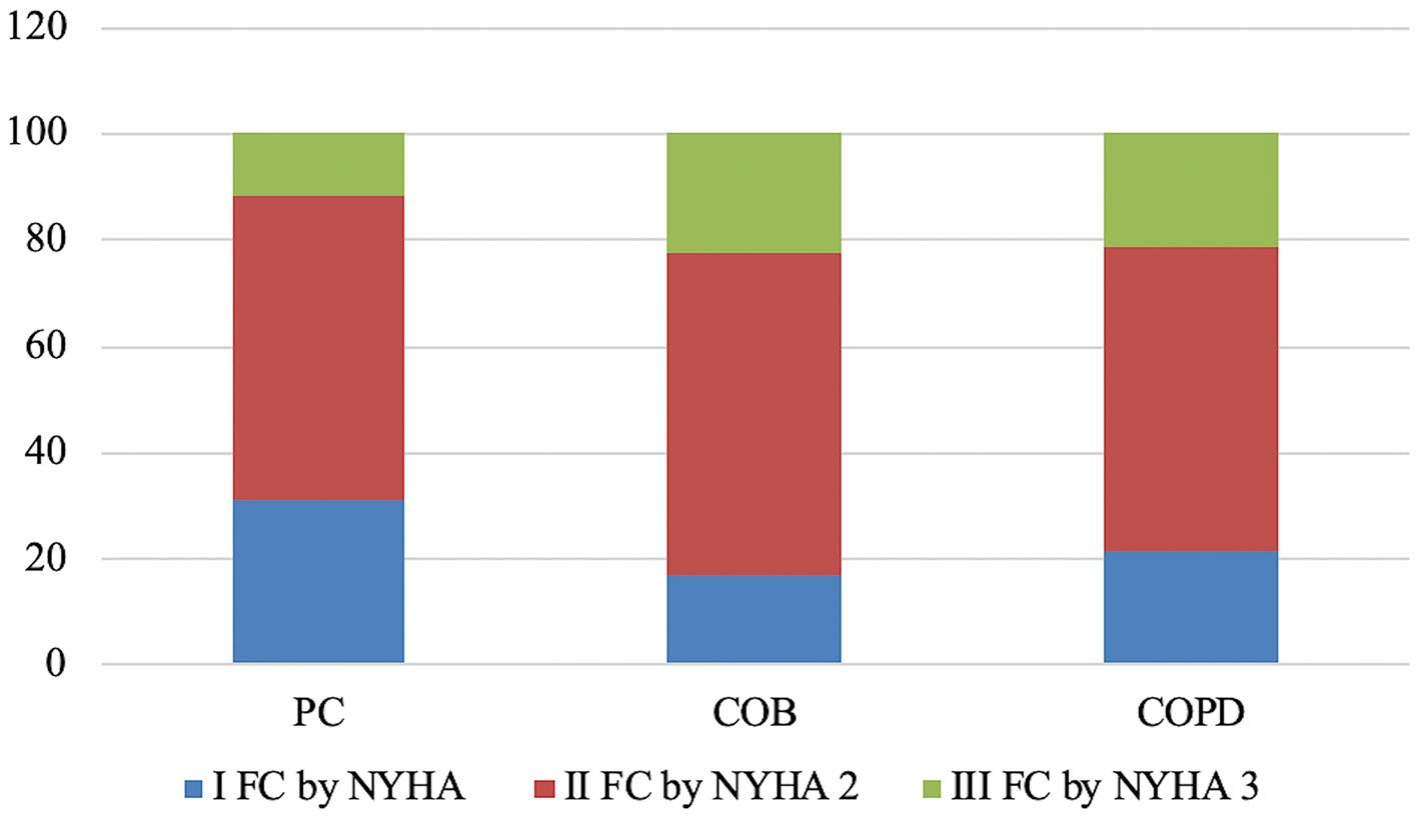
Distribution of patients with chronic cor pulmonale by circulatory failure stage (%). PC, pneumoconiosis; COB, chronic occupational bronchitis; COPD, chronic obstructive pulmonary disease

In the group of 42 patients with PC and pulmonary heart disease, circulatory insufficiency I FC according to the New York Heart Association Functional Classification (NYHA) was revealed in 13 patients (31.0%), II FC in 24 (57.1%) patients, and III FC in 5 patients (11.9%). In 18 patients with pulmonary heart disease in the group of COB patients, circulatory insufficiency I FC was revealed in 3 patients (16.7%), II FC in 11 patients (61.1%), III FC in 4 patients (22.2%). Among 14 COPD patients with this pathology, the findings were as follows: circulatory insufficiency I FC was observed in 3 persons (21.4%), II FC in 8 persons (57.2%), and III FC in 3 persons (21.4%) (Fig. 5).

There was a significant increase of right ventricular anterior wall thickness in patients with dust-induced COB (p < 0.05), COPD (p < 0.01), PC (p < 0.01), which suggests right ventricular hypertrophy in response to increased stress. Authentic increase of the diastolic size of the right ventricle (p < 0.01), a decrease of left ventricular stroke volume (p < 0.05), a decrease of respiratory minute volume (p < 0.05), a decrease of ejection fraction (p < 0.01), shortening fraction (p < 0.05) were observed in all groups. Furthermore, a significant increase of systolic pressure in the pulmonary artery was detected (p < 0.001). The resulting data indicate hypokinetic type formation of central hemodynamics.

Fibroscopic examination of patients with pneumoconiosis showed atrophic processes of the bronchial mucosa in 46 (88.5%), and 6 (11.5%) patients had subatrophic processes. Among patients with COB, mucous membrane atrophy was revealed in 22 (61.1%) patients, subatrophic process in 11 (30.6%), and catarrhal symptoms were observed in 8.3% of cases (3 patients). Atrophy of pulmonary mucosa was stated in 8 COPD patients (30.8%), subatrophic changes of bronchial mucosa were observed in 15 patients (57.7%), and catarrhal changes in 11.5% cases (3 patients).

### Results of radiological and CT examinations

In the group of patients with PC, interstitial changes in the form of linear bands up to 1.5 mm in diameter (gradation “s”) were detected in 30 patients (57.7%). Among them, 2 s-grade was recorded in 22 patients (42.3%), and 1 s-grade in 8 patients (15.4%). Interstitial changes in the form of bands with a diameter ranging from 1.5 to 3.0 mm (gradation “t”) were revealed in 12 patients (23.1%). Of them, 10 patients (19.2%) had 2t-grade and 2 patients (3.8%) had 1t-grade. Nodular changes of 1r-grade were found in 3 patients (5.8%), and nodules with diameters between 1.5 and 3.0 mm of q-grade were revealed in 2 patients. Also, there were two patients with 3q and 2q grade profusion. Radiography revealed no changes in 5 patients (9.6%).

According to CN scans, interstitial changes in the form of linear bands up to 1.5 mm in diameter (gradation “s”) were found in 22 patients (42.3%) with PC. Of them, 2 s-grade was recorded in 17 patients, and 1 s-grade in 5 patients. Interstitial changes in the form of bands with a diameter ranging from 1.5 to 3.0 mm (gradation “t”) were detected in 19 patients (36.5%). Among them, in 2t was stated in 16, and 3t—in 3. Two patients (3.8%) demonstrated changes looking as bands more than 3.0 mm in diameter and grading 3u. Nodules up to 1.5 mm in diameter were found in 4 patients (7.7%) with 1p-grade. Nodular shadows ranging between 1.5 and 3.0 mm in diameter (gradation “q”) were detected in 5 patients (9.6%), with density classified as 1q (3 patients) and 2q (2 patients). Shadows in the form of nodules greater than 3.0 mm in diameter were detected in 2 patients, one each of 2r and 3r grades.

CT method allowed detecting pathological changes in all 52 patients, while X-ray examination in six people showed no signs of lung lesions. CT scanning showed that the number of patients with nodular PC forms (including silicosis) in the studied groups was also increased.

The analysis of X-ray and CT scan data of COB patients showed an insignificant intensification of bronchial vascular pattern that in 92% of patients (according to X-ray data). No pathology was observed in 8% revealed. The diagnosis in this group of patients was made based on the complaints, medical history, physical examination findings, changes in external respiration function indices, fibrobronchoscopy data, sanitary and hygienic features. According to CT findings, bronchial vascular pattern enhancement was detected in all patients. Furthermore, in 19.4% of cases (7 examined patients), its deformity was detected in addition to enhancement.

In the group of patients with established COPD diagnosis, an increased bronchial vascular pattern was noted in 63.7% of cases. At CT investigation, all patients were found to have increased bronchial vascular patterns, with no deformity detected in 5 of them (19.2%).

According to the results of the radiological examination, only 8 cases (15.4%) showed the signs of pulmonary emphysema in the form of widened intercostal spaces, diffuse increase in transparency, or local areas of lung tissue transparency in the group of patients with the diagnosis of PC. The CT diagnostics revealed the signs of emphysema in 38 patients (73.1%). When evaluating CT data of the lungs, small paraseptal air cavities in the apices were revealed in 17 patients, small paraseptal and centrilobular air cavities in 18 patients, multiple small paraseptal air cavities in mediabasal parts of the lungs in 3 patients.

No emphysema was detected in the group of patients with COB during radiological examination. According to CT scans, emphysema was detected in 17 COB patients (47.2%). Of them, small paraseptal and centrilobular air cavities were observed in 14 patients, multiple small paraseptal air cavities in 2, and panlobular areas of decreased lung tissue pneumotization was observed in one patient had.

According to radiological examination, there were no signs of emphysema in the group of patients diagnosed with COPD. They were detected only during lung CT investigation in 10 patients (38.5%) and appeared as small (6 patients) and multiple (4 patients) paraseptal air cavities in the lung apices.

X-ray examination revealed no pathological changes of the pleura in any of the PC patients examined. According to CT scans, pleural thickenings were observed in 28 patients (53.8%). Pleural thickenings up to 5 mm wide (gradation “a”) were registered in 18 patients (45.8%), and pleural thickenings with width from 5 to 10 mm (gradation “b”) in 11 patients.

## Discussion

The problem of optimizing diagnostics of early manifestations of occupational dust-induced lung diseases is caused by the aggravation of initially detected occupational pathology, the predominance of expressed chronic diseases, and the affection of multiple organism’s systems [10, 11]. The specific ratio of industrial aerosol-related conditions with the total number of first-time detections is consistently high [25]. The present study’s findings allow stating that occupational diseases due to exposure to industrial aerosols occupy the third place after diseases due to physical factors, overloads, and stresses in particular organs. The data obtained are in complete agreement with occupational disease statistics in industrial regions of Russia and Aktobe industrial region of the Republic of Kazakhstan [9, 12, 14]. In general, the number of newly identified diseases remained stable throughout the study years (2016–2020). In many cases, the diagnosis is made when the symptoms of the disease become evident, and the patient requests medical assistance himself/herself [14]. Consequently, diseases are often diagnosed when they become fairly serious. The main reason for this is the poor quality of medical checkups, the inadequate optimization of diagnostic measures aimed at the early detection of diseases [7].

The study of clinical symptoms in patients with PC and COB showed that their primary complaint was increased fatigue. Patients with COPD were most often bothered by dry coughing. Dyspnea during physical exercising was noted in all three groups. A major symptom was the reduced work capacity complaint, and patients had to be assessed for the degree of disability. There were no patients without typical complaints in all groups examined. That is, at admission to the Center for Occupational Pathology, clinically developed signs of the disease were already evident upon results of radiography, spirometry, and bronchoscopy. It underlines the need to improve examination procedures for a possible earlier diagnosis of disease.

The framework of this study provided for a clinical and functional examination of patients with diagnosed PC (including silicosis), COB, and COPD to optimize available diagnostic measures.

The spirography study revealed a decrease in FVC indices in COB and COPD patients.

The main spirographic indices of the obstructive syndrome are deceleration of forced expiratory volume in one second due to increased airway resistance and decreased forced expiratory volume (FEV1) and the Tiffeneau index [25, 26]. The drop in the Tiffeneau index (FEV1/FVC) is a more reliable indication of obstructive bronchial syndrome. In patients with PC, the forced expiratory volume in one first second was close to normal, indicating no obstructive changes in the lungs. The main complaints of patients about dyspnea during physical activity and decreased working capacity are related to reduced lung volumes in this group of patients. At the same time, COPD patients experienced a significant decrease in these indexes.

The most sensitive spirographic index indicating the increase of airway resistance is the index of mean forced expiratory flow rate at the level of 25–75% (MEF 25–75%) of FVC [26]. A sharp decrease of peak expiratory flow rate (PEFR) and maximum expiratory flow rate at the level of 25% of FVC can indicate obstruction at a major bronchial level. Furthermore, the values of maximum effective volume in the middle (MEV50%) and at the end of exhalation (MEV75%) also decrease [25].

A relative decrease in MEF25% was observed in the COB group, whereas it was significantly lower than normal in the COPD group. A similar pattern in these groups was observed when recording the peak air volume rate (PEFR) index. No significant dynamics of PEFR were found in patients with PC.

Decrease of expiratory rate indices MEF25%, along with FEV1 and PEFR, is an initial sign of bronchial permeability disturbance and can be registered earlier than clinical symptoms develop. It allows stating latent bronchoconstriction in these patients [23]. The study and analysis of expiratory rate values make it possible to suppose occupational bronchitis at an earlier stage of the process. For the diagnosis of PC, they do not give significant results.

Fibrobronchoscopy is very important in the diagnosis of bronchial disease [25]. The data of the fibrobronchoscopic examinations coincide with the data of other authors on the study of mucosal condition, confirming the prevalence of atrophic processes under the influence of industrial aerosols [27, 28].

The severity of the disease was examined in all patient groups, and complications were determined based on the combination of clinical symptoms and diagnostic studies. Thus, respiratory distress was revealed in 84.6% of all examined patients, and respiratory distress indices corresponded to I degree in 54.6% and II degrees in 30.0% of patients. In all kinds of respiratory distress due to lack of oxygen in the blood and hypoxia, compensating reactions of organs and tissues develop. Long-term chronic respiratory distress leads to the right ventricular heart failure progression due to a lack of oxygen in the heart muscle and constant overload [29].

Following an echocardiological examination, chronic pulmonary heart disease at the decompensation stage was diagnosed in more than half of the patients in each group. The above data indicate that 84.7% of patients with pathological conditions due to exposure to industrial aerosols had complications of the underlying disease in the form of respiratory distress and chronic pulmonary heart disease. The study results confirm that occupational lung disease is more often diagnosed late when the complications result in disability and reduced quality of life [30].

For optimizing early diagnostics of occupational dust-induced diseases of the respiratory tract and lungs, the signs of standard radiological examination and data of lung CT scans were analyzed.

Radiography remains the primary method in diagnosing occupational lung disease due to exposure to aerosols, and only CT is recommended in particular cases [31].

The present study has shown that CT scanning of the lungs makes it possible to detect pathological changes in the lungs, which are usually not detectable by X-ray imaging. The CT method should be used more extensively in the study of respiratory dust-induced diseases. Multispiral CT allows complementing the radiological picture. In particular, it specifies the extent of pathological processes, visualizes interstitial changes of lung tissues, detects emphysema, determines pathological thickening of pleura and pathological changes in bronchovascular pattern, as well as detects elements, which cannot be diagnosed by X-ray analysis.

### Conclusions

The third place in the structure of occupational diseases diagnosed every year in Moscow Center for Occupational Pathology belongs to occupational lung diseases with respiratory distress. A detailed clinical condition was defined when diagnosing lung diseases, including respiratory distress, chronic pulmonary heart disease, and emphysema of the lungs. It attests to the late diagnosis of this group of conditions in the pre-hospital stage. Timely clinical and functional examination (spirography, fibroscopy, echocardiography) of patients during obligatory occupational examinations will allow diagnosing PC (including silicosis), COB, and COPD at an early stage of disease progression.

Multispiral CT of lungs is a highly informative diagnostics method of interstitial diseases of respiratory organs, including PC (2.5 times increased detection of nodular changes in comparison with X-ray method). The CT study of the lungs helps to specify the nature, extent, and profusion of pathological processes in the lung, verify the main diagnosis at the early stages of its progression, and reveal complications of the main diagnosis.

## Data Availability

The datasets used and/or analysed during the current study are available from the corresponding author on reasonable request.

## Abbreviations

PC: Pneumoconiosis
CDB: Chronic dust-induced bronchitis
COPD: Chronic obstructive pulmonary disease
COB: Chronic obstructive (asthmatic) bronchitis
CT: Computed tomography
FVC: Forced vital capacity
FC: Functional class
ACE: Angiotensin-converting enzyme
RF: Respiratory failure
PEFR: Peak expiratory flow rate
NYHA: New York Heart Association
